# 2D Temperature Field Reconstruction Using Optical Frequency Domain Reflectometry and Machine-Learning Algorithms

**DOI:** 10.3390/s22207810

**Published:** 2022-10-14

**Authors:** Alexey Wolf, Nikita Shabalov, Vladimir Kamynin, Alexey Kokhanovskiy

**Affiliations:** 1 Institute of Automation and Electrometry SB RAS, 1 Acad. Koptyug Ave., 630090 Novosibirsk, Russia; 2 Physics Department, Novosibirsk State University, 1 Pirogov St., 630090 Novosibirsk, Russia; 3 Prokhorov General Physics Institute of the RAS, 38 Vavilov St., 119991 Moscow, Russia

**Keywords:** fiber-optic sensor, machine learning, optical frequency domain reflectometry

## Abstract

We present experimental results on the reconstruction of the 2D temperature field on the surface of a 250 × 250 mm sensor panel based on the distributed frequency shift measured by an optical backscatter reflectometer. A linear regression and a feed-forward neural network algorithm, trained by varying the temperature field and capturing thermal images of the panel, are used for the reconstruction. In this approach, we do not use any information about the exact trajectory of the fiber, material properties of the sensor panel, and a temperature sensitivity coefficient of the fiber. Mean absolute errors of 0.118 °C and 0.086 °C are achieved in the case of linear regression and feed-forward neural network, respectively.

## 1. Introduction

Increasing quality of feedback from various industrial plants and buildings requires the collection and processing of large amounts of data. An alternative to point sensors, which are limited in terms of the density of information obtained, are distributed sensors, which able to combine both sensor and data channels. Distributed fiber-optic sensor systems are widely used in the structural health monitoring of materials and industrial objects. Both fiber containing reflective elements (e.g., fiber Bragg grating, or FBG) and unmodified fiber can play the role of the sensor, collecting different-type physical impacts from the environment (temperature, linear strain, bending, humidity, ambient refractive index, etc.) [1,2]. In the latter case, the physical impact is extracted by processing the backscattered Rayleigh, Brillouin, or Raman signals [1,3]. When monitoring relatively compact devices and structures (e.g., elements of robotic systems [4,5], composite materials for the automotive and aerospace industries [6,7,8], etc.) Rayleigh sensor systems based on optical frequency domain reflectometry (OFDR) provide unbeaten performance allowing measuring distributed physical impact on a fiber with a spatial resolution of less than 1 mm [9], and temperature and strain resolution of ±0.1 °C and ±1 με, respectively [10]. In this approach, the frozen inhomogeneities in each individual fiber section act as a random FBG whose spectral shift is converted into temperature/strain change [11]. The exploitation advantages of fiber sensors, including small fiber diameter of ~10–100 microns, immunity to electromagnetic interference, chemical inertness [12], high temperature, and ionizing radiation resistance [7,13,14], along with the high-density distributed sensing provided by an OFDR [15], pave the way to the development of an artificial nervous system for smart cities infrastructure, next-generation vehicles and aircrafts, robotic systems, and assistive systems for people with disabilities, etc.

Growing interest to smart composite materials production opens new areas for fiber sensor application [16,17]. Since an optical fiber acting as a distributed sensor may be embedded in a material with a complex topology, or may pass through several different types of materials, etc., the problem of reconstructing the external physical impact on the material can be significantly complicated. In particular, the user must have sufficient information about the length and trajectory of the fiber, as well as the sensitivity coefficients, at each section of the sensor line. Machine learning (ML) algorithms can be used as an alternative approach for processing data coming from a distributed fiber sensor [18]. To date, they have been used in a number of applications: distributed tactile sensing (including load, position, and pressure area detection) [19,20], pipeline monitoring (including flow, corrosion, and extrinsic intrusion detection) [21,22,23], seismic events detection [24], identifications and classifications of human locomotion [25], etc. Note that the main results in this area have been obtained for methods based on time domain refractometry of the fiber, or on the analysis of the spectral response from a FBG array.

In this paper, we propose a new approach for processing the data obtained from optical frequency domain reflectometer. Using a trained machine-learning algorithm, we directly transform the distributed spectral shift measured in the fiber into the 2D temperature distribution on the surface of the aluminum panel containing the fiber.

## 2. Model of 2D Temperature Sensor

The OFDR technology has shown significant progress in distributed thermometry. Thus, in [26] the temperature measurement accuracy of 0.08 °C with spatial resolution of 5 mm was demonstrated by finding the spectral shift of Rayleigh scattering signal. The measurement accuracy was further improved to 0.001 °C by using an optical fiber containing semi-continuous Bragg gratings [27]. Distributed fiber optic sensors have been proven to operate over a wide temperature range: from a cryogenic temperature of –197 °C with a spatial resolution of 48 cm and an accuracy of 0.34 °C [28] to 850 °C with 1 cm resolution [29]. It was shown in [30] that the method of position-deviation compensation can increase the spatial resolution of the sensor to 0.5 mm when measuring temperatures in the range from 50 to 500 °C; the measurement repeatability is ±0.9 °C in this case.

As a distributed sensor medium we use a Fibercore SM1500(5.3/80)P single-mode optical fiber (protective coating diameter–98 μm, protective coating material–polyimide, mode field diameter–5.3. μm @ 1550 nm, second mode cutoff wavelength–1429 nm, and numerical aperture–0.25). This fiber was not pretreated and did not contain additional resonant structures such as FBGs. The fiber was chosen due to its reduced diameter and relatively high numerical aperture, which reduces optical signal bend losses.

The method for measuring the temperature in an optical fiber is based on measuring the Rayleigh backscattering signal from inhomogeneities frozen into the fiber core. Since the inhomogeneities are randomly distributed along the fiber, their optical spectrum and reflectance vary, depending on the section of the fiber. The distributed measurement of reflection along the fiber yields a reflectogram—a unique “fingerprint” of the fiber, which can then be used to localize and calculate the magnitude of the external impact on the fiber. To obtain such a “fingerprint” we employ OFDR method [31], along with additional post-processing of the received data. The advantage of the OFDR method, as compared to the Raman and Brillouin reflectometry methods, is the unprecedentedly high spatial resolution (~10 µm).

**Figure 1:**
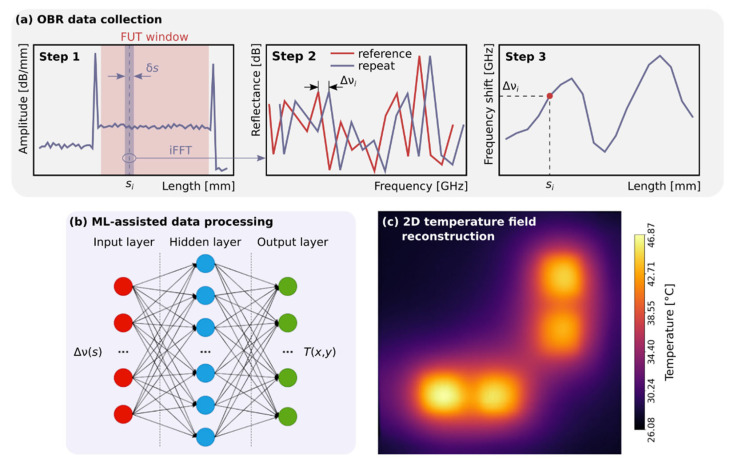
(**a**) Distributed frequency shift measurement for FUT. (**b**) A ML-based model for recalculating the distributed spectral shift Δν(s) into the temperature field T(x, y). (**c**) Reconstructed temperature field.

In our work we use an optical backscattering reflectometer LUNA OBR 4600 [10] (wavelength range–1525–1610 nm, maximum fiber length–30/70 m, sampling resolution–10/20 μm, and temperature–±0.1 °C). Omitting details associated with measurement of the reflectogram and spatially dependent spectrum extraction [32], temperature extraction along the fiber can be reduced to the following steps. First, a reference measurement of the full reflectogram of the fiber under test (FUT) is performed, relative to which all further transformations are made. The fiber section for which the monitoring to be performed (FUT window at Figure 1a) is localized on the reflectogram. Then, the measurement is repeated, after external impact on the FUT, and the FUT window is divided into uniformly distributed spatial segments of width δ*s* located at *s_i_* points, where *i* is the index of the segment (Step 1). Thus, the spatial resolution of a distributed sensor is determined by both the distance between neighboring points Δ*s* = *s_j_* − *s_i_* (or sensor spacing) and the width of the segment δ*s* (or gauge length). For each spatial segment, an inverse discrete Fourier transform is performed, resulting in a frequency representation of the segment (Step 2). If any fiber segment is subjected to external physical impact, the repeated measurement shows the shift of the spectral pattern for this segment. The value of the spectral shift Δν_i_ is determined by calculating the autocorrelation function between the reference and repeated measurements (Step 3). In the absence of another impacts, the temperature at a given point can be calculated as follows [26]:

(1) $$\Delta T_{i}=-\frac{1}{K_{T}}\frac{\Delta \nu_{i}}{\bar{\nu }}=-\frac{\bar{\lambda }}{cK_{T}}\Delta \nu_{i}$$

where *K_T_* is the calibration constants of temperature, $\bar{\nu }$ and $\bar{\lambda }$ are mean frequency and wavelength of the reflectometer scanning range, respectively, and *c* is the light speed.

In our work, we do not track the exact coordinates of the FUT, nor do we perform temperature calibration of the sensor panel; instead, we use ML algorithms that transform the distributed spectral shift of the FUT Δν(*s*) into the temperature field of the sensor panel T(x, y). Figure 1 shows the main steps of the data processing. First, the reflectogram is measured using OBR and the distributed spectral shift along the FUT is calculated (Figure 1a). Then, the distributed spectral shift is fed to the input of a pre-trained ML algorithm (Figure 1b), which performs temperature field reconstruction (Figure 1c). In our case, the algorithm is trained based on 2D temperature fields of the 2D sensor panel captured by a FLUKE RSE600 thermal imager (detector resolution–640 × 480 pixels, frame rate–9 Hz, thermal sensitivity, or noise equivalent temperature difference, –≤0.040 °C at 30 °C).

The following Section 3 gives details on the sensor panel assembly and the data acquisition system. Pre-processing of the data received from OBR and infrared thermal imager, ML algorithms for two-dimensional temperature field reconstruction, and their performance are discussed in Section 4.

## 3. Experimental Setup

**Figure 2:**
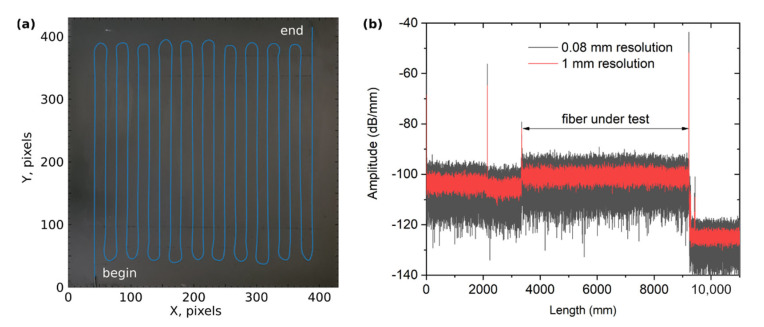
(**a**) Photo of the sensor panel with the integrated FUT (highlighted in blue). The scales correspond to the pixels on the IR thermal imager (1.7 pixels per 1 mm). (**b**) Reflectogram of the FUT measured with a LUNA OBR 4600 after assembly of the panel.

A flat aluminum sheet with a thickness of 1 mm and a size of 250 × 250 mm was used as the base of the sensor panel. To reduce the effects of bending deformations during heating, the panel was fixed to a rigid frame, which, in turn, was pressed against a massive laboratory table. Fibercore SM1500(5.3/80)P fiber was arranged in a serpentine pattern with 10 mm spacing in the central area of the panel, thus filling an area of 200 × 200 mm (Figure 2a). The fiber was fixed to the surface of the panel by cyanoacrylate glue, which was thinly applied to the laid fiber. After the glue dried, a black matte paint was applied to the panel to reduce the effect of glare when measuring temperature with an IR thermal imager. The final assembly was heated at about 80 °C for 2 h to accelerate drying and relieve residual stress associated with the presence of glue and paint. Figure 2b shows the reflectogram of the FUT measured with a LUNA OBR 4600 after assembly of the panel.

**Figure 3:**
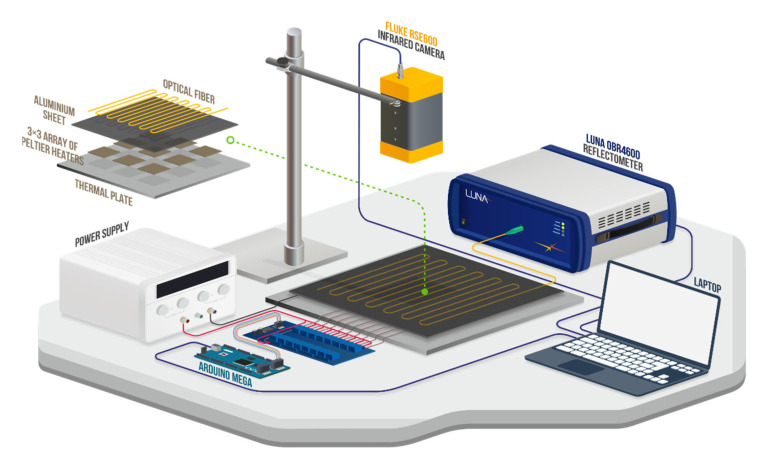
Experimental setup for collecting data from a sensor panel with integrated fiber.

As shown on Figure 3, the panel was mounted on a 3 × 3 array of Peltier elements (square shape, 40 × 40 mm), which, when selectively turned on, heated the panel in a defined area. The cooling backside of a Peltier element was forced against a heat conducting plate for more efficient operation. Thereby, the temperature on the top surface of the sensor panel varied in the range of 20–70 °C at different spatial points. We used a 16-channel relay module and a preprogrammed Arduino Mega 2560 controller, which allowed turning on/off a group of Peltier elements according to a preset algorithm. One measurement cycle consisted of 2 min of heating and 2 min of cooling of the panel. For accelerated cooling the panel was blown with air from the fan installed near the panel.

Simultaneously with heating/cooling of the sensor panel, continuous measurement of FUT reflectograms was performed using LUNA OBR 4600, as well as temperature field on the panel surface using FLUKE RSE600 IR thermal imager. To speed up data acquisition, the reflectometer was switched to a fast wavelength scanning mode, where the central wavelength of the laser source was 1566 nm, the scanning range was 10 nm, and the scanning speed was 100 nm/s, thus providing measurement time of ~0.1 s. The criteria for choosing these parameters were the measurement time comparable with the IR camera, as well as relatively low noise and absence of noise spikes in the distributed frequency shift. The total time for obtaining the distributed frequency shift in the FUT, including reflectogram measurement, signal processing, and data transfer, was ~2 s. The reflectogram measured for the fiber integrated into the sensor panel and held at room temperature was used as a reference when calculating the distributed frequency shift in the FUT.

Synchronization of the devices as well as data accumulation was performed using a laptop, which executed self-written code in the Python language. The code was written using the APIs provided by the manufacturers of the corresponding devices. When synchronizing the devices, we aimed for a minimum delay between the start of measurements on the OBR and IR thermal imager. The full cycle of one measurement and data saving took ~2.5 s; approximately 50 thousand measurements were taken in total.

## 4. Machine Learning Assisted Temperature Field Reconstruction

Machine learning is actively used to reconstruct the temperature distribution of objects on the basis of data obtained using various diagnostic methods (optical, acoustic, hyperspectral analysis, etc.). In [33], support vector machine, random forest, and back propagation neural network were applied to reconstruct the air temperature at the ground surface based on multisource data. The authors of [34] use a kernel regression model and data from acoustic sensors to reconstruct the three-dimensional temperature distribution in a boiler furnace. In [35], two-dimensional temperature fields of laminar flames were reconstructed from infrared hyperspectral measurements with a multi-layer perceptron.

**Figure 4:**
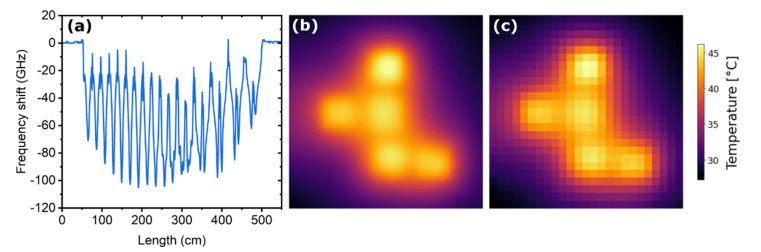
(**a**) Distributed spectral shift of the FUT measured by the OBR. (**b**) Original thermal image with a size of 425 × 425 pixels and (**c**) the corresponding compressed image with a size of 25 × 25 pixels.

As it was mentioned earlier, the application of machine learning simplifies the procedure of the sensor panel calibration, since in this case no input data about the fiber trajectory and the temperature sensitivity of the used materials are required. In our experiment, we investigate the performance of linear regression and a feed-forward neural network (FFNN), which directly relate the distributed frequency shift of the embedded fiber collected by the OBR and the 2D temperature field distribution measured by the thermal imager. We chose linear regression as the first algorithm, assuming that the frequency shift and the temperature of the fiber have a nearly linear relationship. In addition, this algorithm is relatively simple and undemanding to computational resources. A FFNN was chosen because it allows capturing the smallest nonlinear dependencies in data that can affect the result of prediction. At the input, ML algorithms took a distributed spectral shift of the FUT (550 points) collected by the OBR with a gauge length δ*s* of 1 cm and sensor spacing Δ*s* of 1 cm (Figure 4a), and at the output yielded a two-dimensional temperature field distribution with different spatial resolution. The original thermal images of the sensor panel had a size of 425 × 425 pixels corresponding to a single pixel size of 0.588 mm (Figure 4b). A relatively large mismatch between spatial resolutions of the fiber sensor and thermal imager allows to reduce the size of the output data, and thus, to reduce time of a ML algorithm training and evaluation. Therefore, in the training procedure we used both the original thermal images and the compressed ones, with size down to 3 × 3. Figure 4c shows an example of the image compressed down to 25 × 25 pixels. To compress the original thermal images, we applied resampling using pixel area relation. To decompress thermal images predicted by an ML algorithm a bicubic interpolation was used because it gives an optimal combination of processing time and output quality [36].

**Figure 5:**
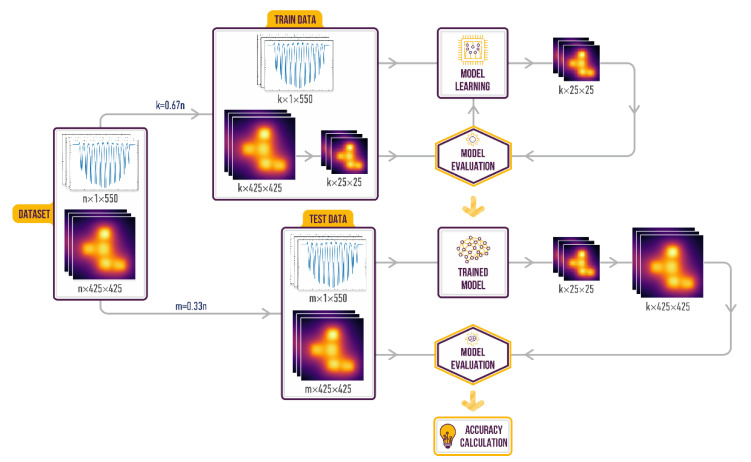
Functional diagram of the training procedure of the ML algorithm reconstructing 2D temperature field of the sensor panel.

The functional diagram illustrating the training procedure of the ML algorithms is presented on Figure 5. First, the experimental dataset containing the distributed spectral shifts and corresponding thermal images was divided into training (67%) and testing (33%) datasets. Training dataset was used to adjust the parameters (e.g., weights and bias) of the ML algorithm. After training, the test dataset was used to assess the performance of an algorithm. Predicted thermal images were decompressed back to 425 × 425 pixels. Finally, original thermal images were compared with the predicted ones. To estimate the prediction error of the ML algorithms we used root mean square error (RMSE) and mean absolute error (MAE) metric functions:

(2) $$RMSE=\sqrt{1/n\sum_{i=1}^{n}{\left(t_{i}-t_{i}^{*}\right)}^{2}},\;MAE=1/n\sum_{i=1}^{n}\left|t_{i}-t_{i}^{*}\right|$$

where $t_{i}^{*}$ is predicted by the algorithm temperature, *t_i_* is an actual temperature measured by the thermal imager, and *n*–size of an image.

**Figure 6:**
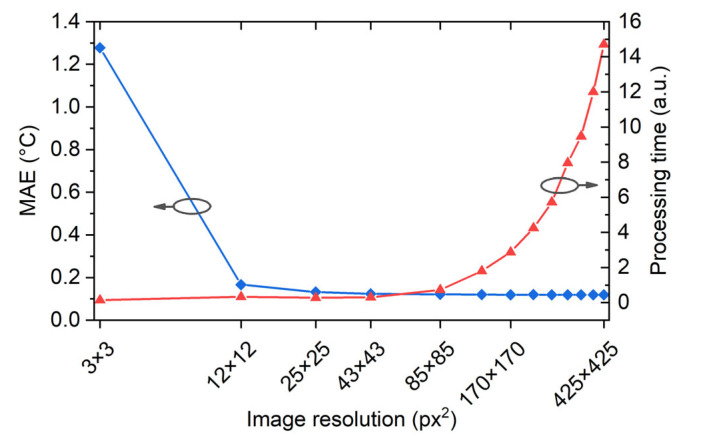
MAE for the linear regression model depending on the size of the input thermal image (blue line). Reconstruction time for a sample depending on the size of the input image (red line).

First, we investigate the performance of a linear regression considering the linear response of the spectral shifts against temperature changes. In this case, the residual sum of squares between the measured values and the target values predicted by the linear approximation is minimized. Figure 6 (black line) demonstrates the MAE of the linear regression model for different sizes of thermal images used for a model training. As one can see, MAE dramatically drops down for images with a resolution of 25 × 25 pixels (compression rate of 17) and becomes almost constant for images with a larger resolution. Note that downsampling of the original image with this rate results in the compressed image whose single pixel corresponds to 1 cm on the panel. On the other hand, computation time for reconstructing a temperature image, that includes the prediction of the compressed image by the ML algorithm and subsequent decompression by the bicubic interpolator, quadratically increases with the size of the image, as Figure 6 (red line) shows. In our case, computational time did not exceed 15 ms on a conventional middle-class CPU. Further, we used image compression to a size of 25 × 25, which is optimal as a balance between spatial precision and computational time. The MAE of linear regression to reconstruct a thermal image was 0.128 °C, which is approximately three times higher than the thermal sensitivity of the used thermal imager.

**Figure 7:**
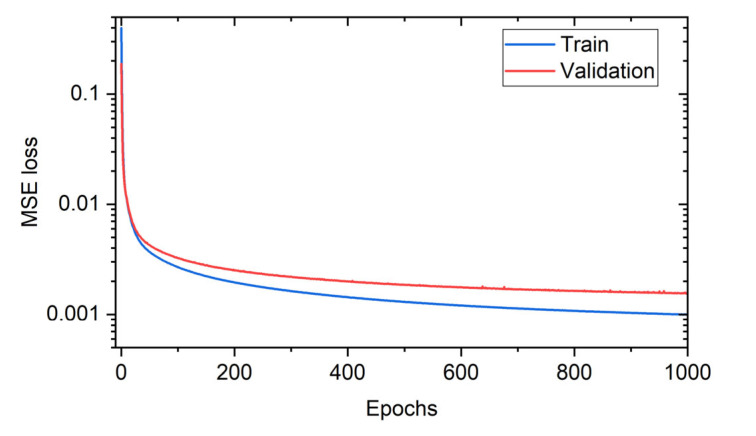
Learning curve of the feed-forward neural network.

Next, we examine the performance of a neural network. The validation dataset (25% of training data) allowed us to tune the network hyperparameters (e.g., topology, size, optimizers, etc.) and to track the model overfitting. The chosen network architecture (FFNN) consists of input, one hidden and output layers, as shown in Figure 1b. All data processing was conducted using the TensorFlow framework in combination with Keras and scikit-learn [37] Python programming libraries. Input layer had 550 neurons corresponding to the size of a distributed spectral shift provided by the OBR. Hidden layer has 2000 neurons with ReLu activation function. These parameters were obtained by a routine hyperparameter optimization procedure with the assistance of the Optuna framework [38]. The output layer has 625 neurons corresponding to the size of a compressed 25 × 25 pixel thermal image. We used RMSprop optimizer for adjusting the weights of the neurons with an initial learning rate of 10^−5^ and mean square error (MSE = RMSE^2^) as loss function. The network was trained during 1000 epochs on the mini-batches with a size of 32. Standardization of the input and the output data was applied by subtracting the mean value and dividing by the standard deviation of the dataset. Learning curve (Figure 7) represents the evolution of the loss function for training and validation datasets (25% of the training dataset) over epochs. It confirms the absence of overfitting the FFNN and the stability of the found architecture.

Table 1 summarizes the performance of the FFNN and linear regression models with and without compression of the thermal images. We relate the better accuracy of the FFNN to its capability to fit nonlinear dependencies between spectral shifts and temperature changes.

In general, the accuracy in reconstructing the physical impact on the sensor depends on a number of factors, including the technical characteristics of the equipment with which the dataset is generated to train ML algorithms, the variability of the external impact on the sensor, the size of the training sample, the algorithm, and the preprocessing of the data. In our measurement system, the main factor that limited the accuracy of the model prediction was the equivalent temperature noise of 40 mK of the IR thermal imager. Although our approach provides high accuracy and speed in temperature field reconstruction based on the distributed frequency shift in the fiber, we can highlight the main drawback of the approach, namely the relatively long data collection process for training the algorithm. One possible solution to this problem is to create a model that will be trained on a limited segment of the sensor panel, on which the variation of temperature occurs.

## 5. Conclusions

Thus, we have shown that the use of machine learning algorithms in processing data from the backscatter reflectometer facilitate the calibration procedure of the sensor panel, since it does not require knowledge about the exact trajectory of the fiber, material properties of the sensor panel, and a temperature sensitivity coefficient of the fiber. At the same time, the proposed approach gives high accuracy in the reconstruction of the temperature field. In this paper, we implemented a protocol for collecting the experimental data needed to train the algorithms, developed models for temperature field reconstruction based on linear regression and FFNN algorithms, and compared their performance under different training conditions. In the case of linear regression, the effect of training data (thermal images) compression on temperature field reconstruction time and prediction accuracy is shown; MAE = 0.118 °C and RMSE = 0.155 °C are achieved in the best case. In the case of FFNN, MAE = 0.086 °C and RMSE = 0.123 °C are achieved by optimizing the neural network architecture.

Although we have considered the relatively simple case of a 2D temperature sensor, we believe that the use of machine learning has high potential in processing data from different types of sensor systems measuring stain, pressure, humidity, refractive index, etc. Thus, machine learning can be useful for multi-parameter sensors, sensors integrated into composite materials and structures with complex topology, sensors with heterogeneous and unknown distribution along the measuring line, increasing spatial accuracy of measurements, and accuracy of measured value recovery.

## Figures and Tables

**Table 1:** RMSE and MAE metrics depending on the used ML algorithm and the thermal image compression rate.

Approach	MAE, °C	RMSE, °C
FFNN with a thermal image compression to 25 × 25 and the output decompression to 425 × 425	0.086	0.123
Linear regression with a thermal image compression to 25 × 25 and the output decompression to 425 × 425	0.128	0.176
Linear regression without thermal image compression/decompression procedure	0.118	0.155

## Data Availability

Data underlying the results presented in this paper are not publicly available at this time but may be obtained from the authors upon reasonable request.
